# Mechanisms Inducing Low Bone Density in Duchenne Muscular Dystrophy in Mice and Humans

**DOI:** 10.1002/jbmr.410

**Published:** 2011-04-20

**Authors:** Anna Rufo, Andrea Del Fattore, Mattia Capulli, Francesco Carvello, Loredana De Pasquale, Serge Ferrari, Dominique Pierroz, Lucia Morandi, Michele De Simone, Nadia Rucci, Enrico Bertini, Maria Luisa Bianchi, Fabrizio De Benedetti, Anna Teti

**Affiliations:** 1 Department of Experimental Medicine, University of L'AquilaL'Aquila, Italy; 2 Ospedale Pediatrico “Bambino Gesù,” Istituto di Ricovero e Cura a Carattere ScientificoRome, Italy; 3 Department of Rehabilitation and Geriatrics, University HospitalGeneva, Switzerland; 4 Muscle Pathology and Immunology Unit, Istituto Neurologico “C. Besta,” Istituto di Ricovero e Cura a Carattere ScientificoMilan, Italy; 5 Department of Internal Medicine, University Hospital “San Salvatore,”L'Aquila, Italy; 6 Metabolic Unit, Istituto Auxologico Italiano, Istituto di Ricovero e Cura a Carattere ScientificoMilan, Italy

**Keywords:** DUCHENNE MUSCULAR DYSTROPHY, OSTEOCLAST, OSTEOBLAST, OSTEOPOROSIS, INTERLEUKIN 6

## Abstract

Patients affected by Duchenne muscular dystrophy (DMD) and dystrophic *MDX* mice were investigated in this study for their bone phenotype and systemic regulators of bone turnover. Micro–computed tomographic (µCT) and histomorphometric analyses showed reduced bone mass and higher osteoclast and bone resorption parameters in *MDX* mice compared with wild-type mice, whereas osteoblast parameters and mineral apposition rate were lower. In a panel of circulating pro-osteoclastogenic cytokines evaluated in the *MDX* sera, interleukin 6 (IL-6) was increased compared with wild-type mice. Likewise, DMD patients showed low bone mineral density (BMD) *Z*-scores and high bone-resorption marker and serum IL-6. Human primary osteoblasts from healthy donors incubated with 10% sera from DMD patients showed decreased nodule mineralization. Many osteogenic genes were downregulated in these cultures, including *osterix* and *osteocalcin*, by a mechanism blunted by an IL-6-neutralizing antibody. In contrast, the mRNAs of osteoclastogenic cytokines *IL6*, *IL11*, *inhibin-βA*, and *TGFβ2* were increased, although only IL-6 was found to be high in the circulation. Consistently, enhancement of osteoclastogenesis was noted in cultures of circulating mononuclear precursors from DMD patients or from healthy donors cultured in the presence of DMD sera or IL-6. Circulating IL-6 also played a dominant role in osteoclast formation because ex vivo wild-type calvarial bones cultured with 10% sera of *MDX* mice showed increase osteoclast and bone-resorption parameters that were dampen by treatment with an IL-6 antibody. These results point to IL-6 as an important mediator of bone loss in DMD and suggest that targeted anti-IL-6 therapy may have a positive impact on the bone phenotype in these patients. © 2011 American Society for Bone and Mineral Research

## Introduction

Duchenne muscular dystrophy (DMD) is an X-linked disorder owing to mutations in the gene encoding dystrophin.(1) It represents the most common muscular dystrophy in children, affecting 1 in 3500 young males.(2) Usually, the first symptom is motor delay, but subsequently, cardiomyopathy and respiratory insufficiency become apparent, leading to death in early adulthood.(3)

In skeletal muscle, dystrophin is localized especially at the sarcolemma, assembled with several other glycoproteins, such as dystroglicans, syntrophins, dystrobrevins, sarcoglycans, and sarcospan, all together making the dystrophin glycoprotein complex, whose integrity is critical for linking the actin cytoskeleton to the extracellular matrix.(4,5) Deficiency of dystrophin expression affects formation of the dystrophin glycoprotein complex, causing disruption of the molecular bridge, with resulting membrane instability, increased susceptibility to mechanical stress, and altered cellular metabolism.(6–10)

Altogether, these events favor myofiber necrosis, which is believed to be the pivotal trigger of the state of inflammation that characterizes skeletal muscle in DMD patients. Indeed, in DMD muscles, a chronic-type mononuclear cell infiltrate is present early, before the onset of muscle weakness,(11) and gene expression profiles show that the inflammatory response dominates the molecular signature of dystrophin-deficient muscles not only in humans but also in the *dystrophin* null mouse, *MDX*, which is the most used murine model for this disease.(12,13) Therefore, a vast body of evidence supports a pivotal role of inflammation in the progression of muscle loss. However, the underlying molecular mechanisms are still unclear.

Consistent with these observations, glucocorticoids, such as prednisone or deflazacort, are the only drugs shown to be effective for DMD treatment, and they delay the progressive loss of muscle strength and function. It is generally believed that their beneficial effects is secondary to their potent anti-inflammatory action. However, long-term treatment with glucocorticoids exposes patients to severe side effects, particularly on the skeletal system.(14–17)

Involvement of the skeletal system in DMD is very common. Osteoporosis is observed in many patients, and up to a third of them are affected by pathologic fractures of both long bones and vertebrae.(18,19) Fractures have a significant impact on mobility and quality of life of patients, but the mechanisms underlying the involvement of bone tissue in DMD are still poorly understood. The literature is scarce of data related to bone mass and metabolism in DMD patients,(20,21) and it is still unknown whether DMD by itself leads to a reduced accrual of bone mineral density (BMD) independent of glucocorticoid therapy.

In this study, we analyzed the bone phenotype of the *MDX* mouse and evaluated the ability of sera collected from DMD patients before the onset of glucocorticoid therapy and from *MDX* mice to modulate osteoblast and osteoclast function. We report that a cytokine imbalance appears to contribute to the bone loss in DMD and point to interleukin 6 (IL-6) as a possible systemic mediator of the damage induced by chronic inflammation in bone, which could be subjected to therapeutic neutralization.

## Materials and Methods

### Animals

*MDX* (X chromosome-linked muscular dystrophy) homozygous mice (CB6F1/C57BL6 background) carrying a spontaneous single-base mutation on exon 23 of the *dystrophin* gene(22) were used for analysis of the bone phenotype at 6 months of age. Neonatal CD1 mice were used to isolate calvarial bones for organ cultures.

Procedures involving animals and their care were conducted in conformity with national and international laws and policies (European Economic Community Council Directive 86/609, Italian Legislative Decree 116/92, National Institutes of Health Guide for the Care and Use of Laboratory Animals) and were approved by our internal ethical board.

Mice were euthanized by cervical dislocation, and long and parietal bones were removed, cleared of soft tissues, and processed for micro–computed tomography (µCT), histology, histomorphometry, and reverse-transcriptase polymerase chain reaction (RT-PCR) analysis or for organ cultures.

### Assessment of trabecular and cortical microarchitecture

µCT (µCT40; Scanco Medical, Basserdorf, Switzerland) was used to assess trabecular bone volume fraction [bone volume/total volume (BV/TV)] and microarchitecture in the metaphyseal region of the tibia and cortical geometry at the midtibia. For trabecular bone, the BV/TV (%), the trabecular thickness (µm), the trabecular number [number of plates per unit of length (mm)], and the trabecular space (µm) were assessed on 100 contiguous µCT slides starting 100 slides below the growth plate. For cortical bone, the average total area (TA) inside the periosteal envelope (mm^2^), the bone area (BA) within this same envelope (mm^2^), the marrow area (MA, mm^2^), and the average cortical thickness (mm) were assessed at 6-µm resolution on 54 contiguous µCT slides.

### Bone histology and histomorphometry

Tibias and parietal bones were fixed in 4% formaldehyde in 0.1 M phosphate buffer (pH 7.2), dehydrated in acetone, and processed for glycol-methacrylate embedding without decalcification. Histomorphometric measurements were carried out on 5-µm-thick sections with an interactive image-analysis system (IAS 2000; Delta Sistemi, Rome, Italy) as described previously,(23,24) and with the suggested nomenclature.(25) Osteoclast number/bone surface (*n*/mm^2^) and osteoclast surface/bone surface (%) were evaluated after staining the sections for tartrate-resistant acid phosphatase (TRACP). Osteoblast surface/bone surface (%) was evaluated after staining the sections with methylene blue/azure II. Finally, dynamic assessment of the mineral apposition rate (MAR) was calculated after double injection of calcein 10 and 3 days before euthanization. All reagents were from 2M Strumenti (Rome, Italy) and Bio Optica (Milan, Italy).

### Patients

The study was approved by the ethical committees of Ospedale Pediatrico Bambino Gesù, Rome, Istituto Auxologico Italiano, Milan, Istituto Neurologico Besta, Milan, and University Hospital San Salvatore, L'Aquila, Italy. Patients were diagnosed and followed at Ospedale Pediatrico Bambino Gesù, Istituto Auxologico Italiano, and Istituto Neurologico C. Besta, and their characteristics and clinical parameters are shown in Table 1. The study was conducted with the informed consent of healthy donors and patients' parents and involved collection of human biologic samples (ie, peripheral blood, serum, urine, and bone fragments) and dual-energy X-ray absorptiometry (DXA). We strictly adhered to all current ethical and safety provisions applicable, in compliance with the Declaration of Helsinki in its latest version and the regulation of our competent ethical committees.

**Table 1:** Characteristics of the Studied Subjects

Subjects	*N*	Age	Weight	Height
Controls	11	8.7 ± 2.7	26.7 ± 2.02	124.0 ± 6.7
Patients	16	8.4 ± 1.9	17.7 ± 3.8	103.0 ± 7.2

*Note:* Data are expressed as means ± SD.

All DMD subjects recruited for this study were 5- to 10-year-old males. Diagnosis was based on clinical data and on molecular, morphologic, and immunochemical evaluation confirming the absence of dystrophin in muscle fibers. Patients were not treated with any glucocorticoid therapy and were not affected by other diseases that could influence bone metabolism. Control subjects were matched for age and gender and tested for standard serum markers to exclude any inflammatory status.

### Bone mass

BMD was evaluated by DXA (Hologic Discovery A in Milan and Hologic Delphi W in Rome; Hologic, Inc., Waltham, MA, USA) at the lumbar spine (L_2_–L_4_) using the same scanning and analysis protocol in all centers. A standard adjustment, based on the approximate bone volume calculated, considering lumbar vertebral bodies as cylinders, was used. This adjustment gives a measure called *bone mineral apparent density* (BMAD).(26,27) BMAD was calculated and expressed as a *Z*-score. The *Z*-score was calculated, selecting a reference sample of age-matched healthy Italian boys from the database of the Istituto Auxologico Italiano.

### Serum markers

Patients' serum total calcium, phosphorus, bone turnover markers, and calciotropic hormones were measured using standard methods. Radioimmunologic assay kits were used to measure serum osteocalcin (01051950; TechnoGenetics, Milan, Italy) and 25-hydroxyvitamin D (310600; Stillwater, MN, USA). ELISA kits were used to measure urinary N-terminal telopeptide of procollagen type 1 (NTX; Ostex Intern, Seattle, WA, USA) and serum bone alkaline phosphatase (BALP; EIA, Metra BAP, Quidel, San Diego, CA, USA). Immunoradiometric assay kit was used to measure N-parathyroid hormone (PTH; 01052410; TechnoGenetics). Radioreceptor assay was used to measure 1,25-dihydroxyvitamin D_3_ (3520; Nichols Institute Diagnostic, San Juan Capistrano, CA, USA). ELISA kits were used to measure osteoprotegerin (OPG), serum receptor activator of NF-κB ligand (sRANKL; kits RD194003200 and RD193004200R; BioVendor, Candler, NC, USA), as well as interleukin 6 (IL-6), tumor necrosis factor α (TNF-α), IL-12 p70, IL-11, inhibin-βA, and transforming growth factor β2 (TGF-β2) (HS600B, HSTA00D, DY1270, D1100, DAC00B, and DB250; R&D Systems, Minneapolis, MN, USA).

### Reagents for the in vitro and animal studies

Cell culture medium, fetal calf serum (FCS), reagents, and Trizol for RNA extraction were from Invitrogen (Carlsbad, CA, USA). Sterile glassware was from Falcon Becton Dickinson (Meylan, France). Cytokines for cell cultures and the blocking antibody against human and mouse IL-6 were from Pepro-Tech (Rocky Hill, NJ, USA). Unless otherwise specified, all other reagents were of the purest grade from Sigma-Aldrich (St Louis, MO, USA).

Mouse serum C-terminal collagen type 1 cross-links (CTX) and TRACP activity were evaluated in serum and/or in conditioned medium by ELISA kits from Pantec (AC06F1 and SBTR103, Turin, Italy), whereas IL-6, sRANKL, and OPG were measured by ELISA kits from R&D Systems (M6000B, MTR00, and DY459).

### Human primary osteoblast cultures

Bone fragments were obtained from healthy subjects who underwent femoral surgery for traumatic fractures. Bone fragments were subjected to sequential digestion with 1 mg/mL of *Clostridium histolyticum* type IV collagenase and 0.25% trypsin as described previously.(28) Cells obtained with this method were positive for alkaline phosphatase (ALP) activity and expressed the osteoblast markers PTH/PTH-related peptide receptor, type I collagen, osteocalcin, osteopontin, bone sialoprotein II, and Runx2.(28) Osteoblasts were plated in 6-well multiplates, grown to 80% confluence, then starved for 24 hours in medium with 1% FCS, and subsequently treated with medium containing 10% human serum from DMD patients or from healthy donors for 48 hours, in agreement with previous work.(29)

### Nodule mineralization

Human osteoblasts were grown until 80% confluence. Medium was replaced with mineralization medium composed of Dulbecco's modified Eagle medium (DMEM), 10 mM β-glycerophosphate, 50 µg/mL of ascorbic acid, and 10% serum from either healthy donors or DMD patients. Medium was replaced every 3 days, and cells were cultured for 3 weeks. At the end of incubation, mineralization was detected by von Kossa staining.

### ALP activity

ALP activity was evaluated biochemically using Sigma Kit N1891 or, otherwise, histochemically using Sigma Kit 85L3R in cultured osteoblasts previously fixed in 4% paraformaldehyde in 0.1 M cacodylate buffer.

### Osteoclast preparation from peripheral blood monocytes

Osteoclast precursors were isolated from human peripheral blood from DMD patients and healthy donors as described previously(29) and cultured for 14 days in standard medium containing 10% FCS in the presence of 25 ng/mL of macrophage colony-stimulating factor (M-CSF) and 30 ng/mL of sRANKL. Cells from healthy donors also were incubated for the same time in medium containing 10% human serum from DMD patients or healthy donors in the presence of 25 ng/mL of M-CSF and a suboptimal concentration of sRANKL (0.5 ng/mL).(29) Medium and factors were replaced every 3 days.

### TRACP activity assay

Cells were fixed in 4% paraformaldehyde in 0.1 M cacodylate buffer for 15 minutes and washed in the same buffer. TRACP activity was detected histochemically using Sigma-Aldrich Kit 387-A according to the manufacturer's instruction.

### Comparative real-time RT-PCR

Total RNA was extracted using the Trizol procedure; then 1 µg was reverse transcribed, and the equivalent of 0.1 µg was employed for the PCR reactions using the Brilliant SYBR Green QPCR Master Mix (Aurogene, Rome, Italy). Primer sequences and real-time PCR conditions are listed in Supplemental Table S1.

### cDNA real-time array

Total RNA was extracted from human osteoblasts cultured for 48 hours in the presence of 10% serum from DMD patients or from healthy subjects and employed for the real-time PCR array. Two sets of arrays were used: a panel of osteogenic markers (human osteogenesis PCR array PAHS-026; Superarray Biosciences, Frederick, MD, USA) and a panel of inflammatory cytokines (human cytokines PCR array PAHS-021, Superarray Biosciences). Briefly, c-DNA from osteoblasts was mixed with a SYBR Green Master Mix (RT^2^ SYBR Green qPCR master mix; PA-012; Superarray Biosciences) and then dispensed in the wells for the specified panels. Wells were subjected to real-time PCR (Stratagene MX 3000, La Jolla, CA, USA) following the manufacturer's instructions.

### Array data analysis

Array data were automatically analyzed by the dedicated software, RT^2^ Profiler PCR Array Data Analysis Template Version 3.2 (Superarray Biosciences). Briefly, the gene expression profiles from each array were normalized versus a set of 6 housekeeping genes. Genes from the two sets of data then were distributed by the threshold cycle values, plotted by two-tailed Student's *t* test that assumes a normal distribution, and filtered by a significance of *p* < .05.

### Calvarial organ cultures

Calvaria from 4-day-old CD1 mice (Harlan Laboratories, Udine, Italy) were explanted, dissected free of adjacent connective tissue, and placed in α modified essential medium (α-MEM) supplemented with 10% sera from 6-month-old wild-type or *MDX* mice and incubated with 1 µg/mL of blocking antibody against IL-6 or with irrelevant IgG as control. After 7 days, conditioned medium was collected for ELISA assay, and bones were fixed and processed as earlier for histomorphometric analysis.

### Statistics

Data distribution was analyzed previously by the Anderson-Darling test. If normally distributed, data were expressed as the mean ± SD of at least three independent experiments or three animals per group; otherwise, they were expressed as a value range. Statistical analysis was performed by one-way analysis of variance, followed by Student's unpaired *t* test for the normally distributed data and by the Wilcoxon test in the other cases. *p* Values of less than .05 were considered statistically significant.

## Results

### Bone phenotype in *MDX* mice

**Fig. 1:**
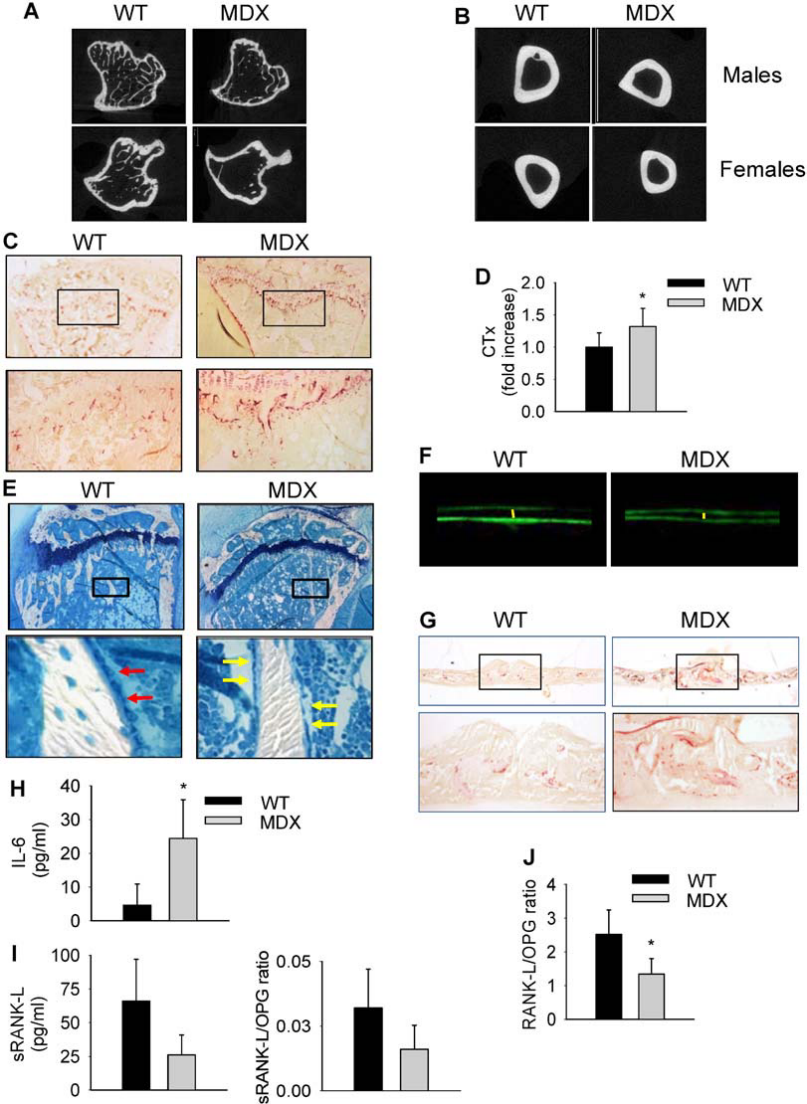
Bone phenotype in *MDX* mice. µCT analysis performed (*A*) in the tibial proximal spongiosa and (*B*) in cortical bone of the tibial midshafts of wild-type and *MDX* male and female animals. (*C*) Histochemical detection of the osteoclast-specific marker TRACP (*purple*) in proximal tibias of wild-type and *MDX* mice. Original magnification ×2.5 (*upper panels*) and ×10 (*lower panels*). (*D*) Detection of C-terminal telopeptide of type 1 collagen (CTX) in *MDX* sera by ELISA, as specified in “Materials and Methods.” Average value in wild-type mice was 24.5 ± 7.8 ng/mL. *p = .04 versus wild type. (*E*) Histologic sections of secondary proximal spongiosa of wild-type and *MDX* mouse proximal tibias stained with methylene blue/azure II. Original magnification ×2.5 (*upper panels*) and ×40 (*lower panels*). *Red arrows* = active cuboidal osteoblasts; *yellow arrows* = inactive flat osteoblasts. (*F*) Calcein (*green fluorescence*) labeling of secondary proximal spongiosa from wild-type and *MDX* mice showing the trabecular mineral apposition (distance between the two fluorescent labels, evidenced by the yellow lines). Original magnification ×40. (*G*) Coronal sections of calvaria from wild-type and *MDX* mice stained for histochemical detection of TRACP (*purple*). Original magnification ×2.5 (*upper panels*) and ×10 (*lower panels*). (*H*) Detection of IL-6 in wild-type and *MDX* sera by ELISA. (*I*) Detection of sRANKL in wild-type and *MDX* sera by ELISA. OPG was unchanged in the two genotypes, whereas RANK-L/OPG ratio was reduced in *MDX* sera and also shown in panel *I*. *p = .04 versus wild type. (*J*) RNA was extracted from femurs of 6-month-old *MDX* and wild-type mice and reverse transcribed; then cDNA was subjected to comparative real-time PCR using primer pairs and conditions specific for *RANKL* and *OPG.* RANK-L/OPG ratio is shown. *p = .003 versus wild type. Values are normalized versus the house keepinggene *GAPDH*. All values are the mean ± SD of at least four sera samples or three animals per group.

In *MDX* mice of both genders, significant bone loss was apparent at trabecular and cortical levels versus wild-type mice. In particular, µCT analysis showed a significantly lower bone volume percentage of the tibial metaphysis in *MDX* animals (Table 2, Fig. 1*A*). At the tibial midshaft, there was an obvious thinning of the collar in *MDX* animals compared with wild-type animals (Table 2, Fig. 1*B*), with a consequent lower cortical total area and bone area in both genders as well as a lower cortical thickness and marrow area in females (Table 2). These observations demonstrate that the loss of dystrophin causes an osteopenic phenotype in the *MDX* mouse model. Therefore, we investigated the underlying cellular mechanism by examining osteoclasts and osteoblasts in vivo.

**Table 2:** Bone Microarchitectural and Histomorphometric Parameters in *MDX* Mice

	Males			Females		
		
	Wild type	*MDX*	*p* Value	Wild type	*MDX*	*p* Value
Trabecular bone, µCT^a^						
Bone volume/total volume, %	9.2 ± 0.9	5.7 ± 1.1	.04	5.8 ± 0.7	3.5 ± 1.5	.04
Trabecular number^b^	4.10 ± 0.11	3.5 ± 0.3	.02	3.0 ± 0.9	2.8 ± 0.9	.23
Trabecular thickness, µm	36.0 ± 2.3	37.0 ± 2.6	.67	42.1 ± 2.2	42.9 ± 4.0	.72
Trabecular space, µm	236.0 ± 7.3	289.0 ± 28.5	.04	320.0 ± 25.0	360.0 ± 46.0	.27
Cortical bone, µCT						
Cortical total area, mm^2^	0.62 ± 0.04	0.55 ± 0.02	.03	0.57 ± 0.04	0.46 ± 0.05	.02
Cortical bone area, mm^2^	0.40 ± 0.01	0.35 ± 0.02	.04	0.37 ± 0.03	0.30 ± 0.02	.03
Cortical marrow area, mm^2^	0.22 ± 0.03	0.20 ± 0.01	.23	0.20 ± 0.01	0.16 ± 0.02	.02
Cortical thickness, mm	244.0 ± 13.0	230.0 ± 11.0	.23	238.5 ± 6.9	222.0 ± 7.2	.04
Trabecular bone, histomorphometry^c^						
Osteoclast surface/bone surface, %	15.9 ± 4.4	27.4 ± 3.7	.03	11.2 ± 4.3	21.2 ± 4.2	.009
Osteoclast number^d^	12.8 ± 1.2	21.5 ± 2.5	.006	10.1 ± 3.1	15.0 ± 3.0	.05
Osteoblast surface/bone surface, %	28.9 ± 8.5	19.6 ± 1.9	.05	36.6 ± 3.5	20.4 ± 3.5	.005
Mineral apposition rate, µm/d	Not measured	Not measured		0.60 ± 0.16	0.45 ± 0.13	.03
Calvarial bone, histomorphometry^e^						
Bone volume/total volume, %	78.7 ± 2.1	73.3 ± 2.1	.03	79.0 ± 4.9	68.4 ± 3.4	.04
Osteoclast surface/bone surface, %	2.2 ± 1.3	3.8 ± 1.1	.20	15.3 ± 5.1	19.7 ± 3.1	.18
Osteoclast number^d^	1.5 ± 0.8	3.4 ± 0.4	.02	10.9 ± 2.8	15.0 ± 1.8	.04
Osteoblast surface/bone surface, %	6.2 ± 0.9	5.4 ± 1.7	.51	7.8 ± 2.6	7.9 ± 2.3	.93

*Note:* Values are the mean ± SD of at least 3 animals per group. *MDX* = X chromosome-linked muscular dystrophy.

a Proximal tibia metaphysis.

b Number of plates per unit of length (mm).

c Proximal tibia metaphysis, 100 µm from distal end of growth plate excluding the endocortical surfaces, longitudinal sections.

d Number of osteoclasts per mm^2^ of bone surface.

e Coronal sections.

Histochemical analysis showed more intense staining for the osteoclast-specific marker TRACP lining the growth plate mineralized cartilage and bone trabeculae in the proximal spongiosa of the tibias of *MDX* animals (Fig. 1*C*). Higher magnification showed more osteoclasts in *MDX* mice than in wild-type mice (Fig. 1*C*), and histomorphometry confirmed higher osteoclast surface and osteoclast number per bone surface in the secondary spongiosa of *MDX* versus wild-type mice in both genders (Table 2). Consistent with the histologic results, CTX, a marker of in vivo bone resorption, was significantly higher in *MDX* mice (Fig. 1*D*). Staining of bone sections of *MDX* mice with methylene blue/azure II demonstrated that the bone trabecular surface was especially coated by flattened and presumably less active osteoblasts than in wild-type mice (Fig. 1*E*) and that the osteoblast surface per bone surface was lower in *MDX* than in wild-type mice in both genders (Table 2). To investigate dynamically whether osteoblast deficiency had an impact on ossification, we evaluated the mineral apposition rate (MAR) by in vivo double fluorescent calcein labeling of trabecular bone. A 25% less MAR was found at the proximal secondary spongiosa of *MDX* mice versus wild-type mice (Table 2, Fig. 1*F*).

To distinguish between humoral and mechanical influence on bone structure, we evaluated calvarial bone histology and histomorphometry because these bones are modestly affected by mechanical strength. Histomorphometry showed lower bone volume percentage (Table 2) and higher number of osteoclasts (Table 2, Fig. 1*G*) in *MDX* compared with wild-type mice.

Interestingly, in a panel of circulating pro-osteoclastogenic cytokines evaluated in the sera, IL-6 was found to be increased in *MDX* mice (5.3-fold) compared with wild-type mice (Fig. 1*H*). Surprisingly, and in contrast with the increased osteoclast activity observed by histomorphometry, sRANKL and, consequently, the sRANKL/OPG ratio were not increased but rather decreased, although not significantly (*p* = .07 and *p* = .1, respectively; Fig. 1*I*) in *MDX* compared with wild-type mice. Similar results were observed when the *RANKL/OPG* mRNA ratio was examined in the femurs of *MDX* compared with wild-type mice (Fig. 1*J*).

### Bone density and cytokine imbalance in DMD patients

**Fig. 2:**
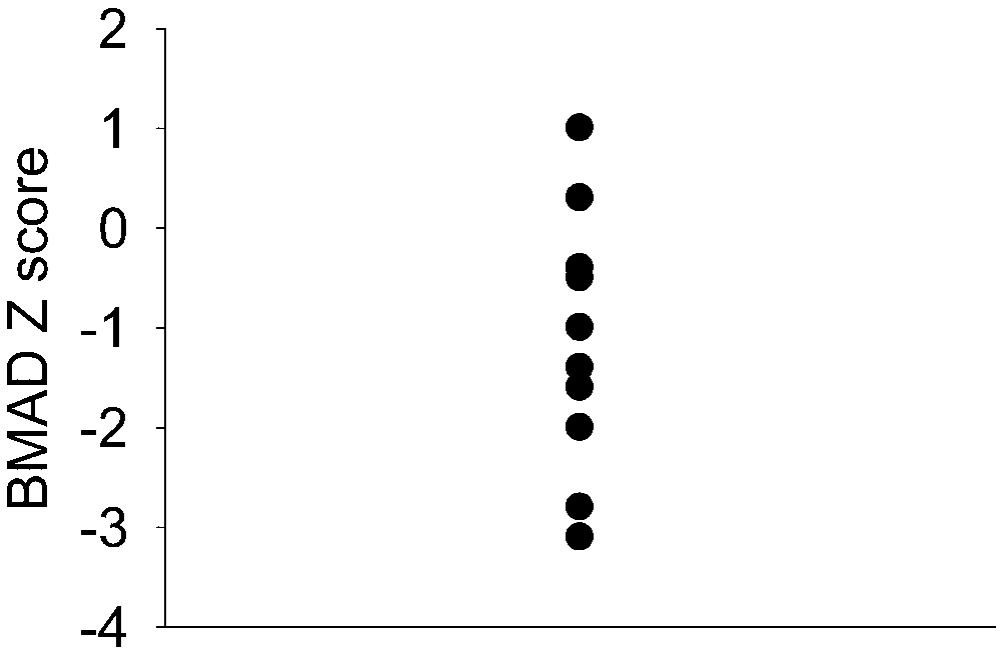
Distribution of the 16 DMD patients along the bone mineral apparent density (BMAD) *Z*-score gradient.

In agreement with our observations in mice, among the bone turnover parameters evaluated in 16 patients with DMD before the onset of treatment with glucocorticoids (Table 3), *NTX*, a marker of bone resorption, was higher than normal. Consistently, the BMAD *Z*-score was lower than normal. The distribution plot in Fig. 2 shows that in 6 patients (37%) the BMAD *Z*-score was lower than −1. In particular, in 3 of them it was between −1.1 and −2, and in the other 3 it was below −2.

**Table 3:** Bone Turnover Parameters in Sera of DMD Patients

Marker	DMD patients	Normal ranges
Serum Ca (mg/dL)	10.0 ± 0.37	8.4–10.4
Serum P (mg/dL)	5.4 ± 0.49	3.5–5.5
Serum Mg (mg/dL)	2.3 ± 0.17	1.6–2.9
Serum Cr (mg/dL)	0.24 ± 0.07	0.2–1.0
Urinary NTX (nMBCE/mMCR)	498 ± 139	60–380
Serum OCN (ng/mL)	60 ± 14	5–76
Serum BALP (U/L)	63 ± 3.8	14.2–72.7
Serum PTH (pg/mL)	31 ± 14	20–59
Serum 25(OH)D (ng/mL)	20 ± 15	20–60
Serum 1,25(OH)_2_D_3_ (pg/mL)	36 ± 12	26–49

*Note:* Data are expressed as means ± SD. Ca = calcium; P = phosphorus; Mg = magnesium; Cr = creatinine ; BALP = bone alkaline phosphatase; OCN = osteocalcin; PTH = parathyroid hormone; 25(OH)D = 25-hydroxivitamin D; 1,25(OH)_2_D_3_ = 1,25-dihydroxivitamin D_3_.

To assess the involvement of circulating cytokines, our 16 DMD patients and 11 gender- and age-matched healthy controls were analyzed by ELISA for the serum concentrations of a panel of cytokines. Notably, IL-6 showed a significant increase in DMD patients compared with healthy subjects (Table 4), consistent with their low BMAD. sRANKL was 54% lower in DMD sera, and the sRANKL/OPG ratio was significantly lower in the DMD group compared with the healthy group. This is similar to our observations in *MDX* mice and in sharp contrast with the low *Z*-scores and high urinary NTX levels observed in the patients.

**Table 4:** Serum Cytokine Levels in DMD Patients

Cytokine	Healthy subjects	DMD patients	*p* Value
IL-6, pg/mL	1.93 ± 1.38	3.77 ± 2.71	.04
TNF-α, pg/mL	Not detectable	Not detectable	—
IL-12 p70, pg/mL	Not detectable	Not detectable	—
sRANKL, pM	51–457^a^	10–302^a^	.007
OPG, pM	3.4 ± 1.3	4.0 ± 1.3	NS
sRANKL/OPG	15–163^a^	1.8–107^a^	.005

*Note:* Data are expressed as means ± SD or as a range. *p* Value indicates the significance level. NS = not significant.

a These data are not normally distributed, as evaluated by the Anderson-Darling test. Therefore, they are expressed as value ranges.

### Effect of DMD sera on osteoblasts

To address whether imbalance of circulating cytokines can be involved in the pathogenesis of bone loss in DMD, we analyzed the effects of DMD sera in cell cultures. To reduce the effects of variability, pools of equivalent aliquots of the sera from the 16 DMD patients and the 11 control subjects described in Table 1 were used. In these pools, IL-6 concentration was measured and found to be 4.0 ± 0.4 pg/mL in DMD and 1.6 ± 0.1 pg/mL in control sera (*p* = .02).

**Fig. 3:**
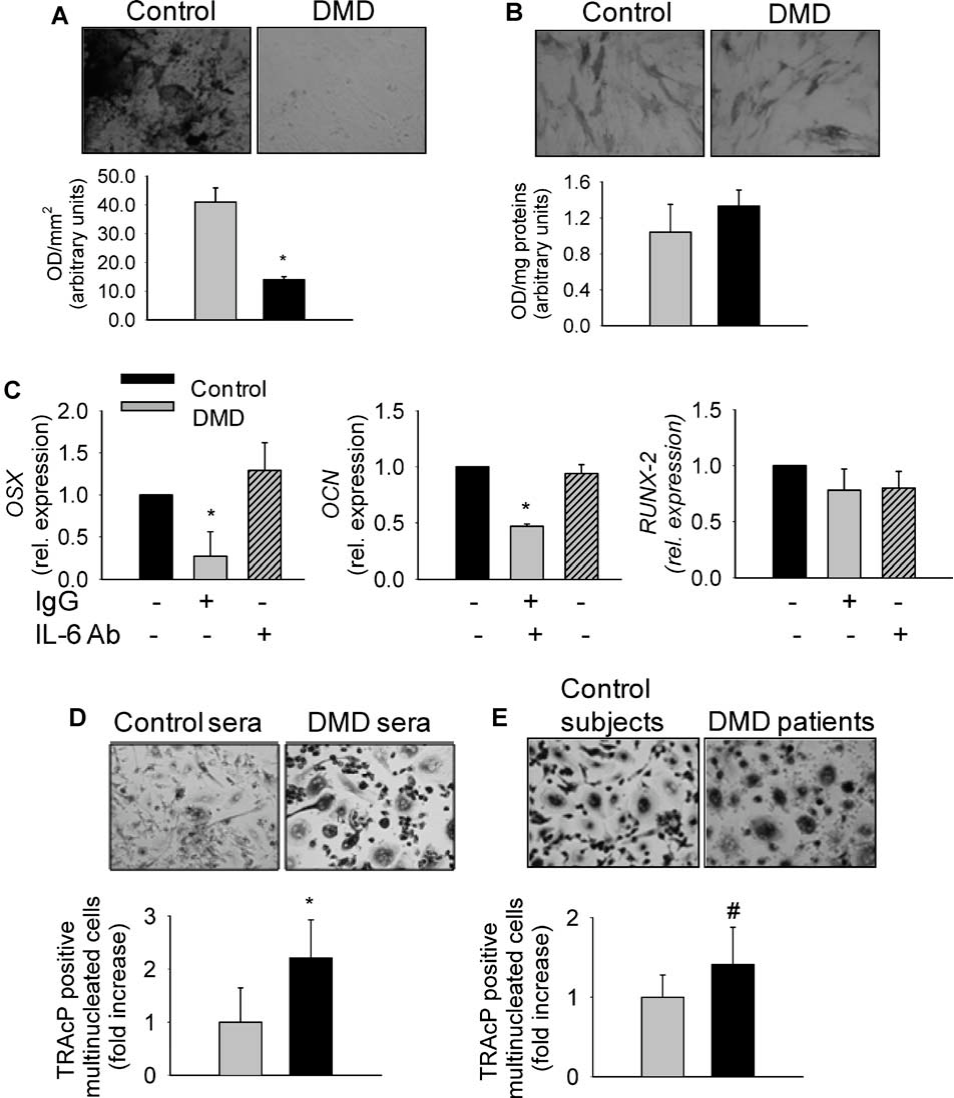
Effects of sera from DMD patients on in vitro human bone cells. (*A*) Osteoblasts were cultured for 3 weeks in the presence of 10% sera pooled from healthy donors (Control) or DMD patients and with ascorbic acid and β-glycerophosphate, as described in “Materials and Methods,” to favor mineralization. Cultures then were fixed and stained to reveal mineralized nodules by the von Kossa reaction (*dark staining*, *panels*). Intensity of mineralization was measured by densitometry (*graphs*). *p = .0005 versus control. (*B*) Osteoblasts were grown as above and then evaluated for alkaline phosphatase (ALP) activity by histochemical detection (*panels*, *dark staining*) or by biochemical detection (*graphs*). (*C*) Osteoblasts were grown in the presence of 10% sera pooled from healthy controls or DMD patients, with an IL-6-blocking antibody (IL-6 Ab) or an irrelevant IgG, as indicated. After 48 hours, RNA was extracted and reverse transcribed, and cDNA was subjected to comparative real-time PCR using primer pairs and conditions specific for *osterix* (*OSX*), *osteocalcin* (*OCN*) and *Runt-related transcription factor 2* (*RUNX2*). ^#^*p* = .004 and *p = .0005 versus control. (*D*) Human peripheral blood mononuclear cells from healthy donors were cultured in the presence of 25 ng/mL of M-CSF, 0.5 ng/mL of sRANKL, and 10% sera from healthy donors (Control) or DMD patients and evaluated for TRACP positivity, as described in “Materials and Methods.” *p = .003 versus control. (*E*) Human peripheral blood mononuclear cells from control subjects or 3 DMD patients were cultured in the presence of 10% FBS, 25 ng/mL of M-CSF, and 30 ng/mL of sRANKL and evaluated for TRACP positivity, as described in “Materials and Methods.” ^#^*p* = .05 versus control. Average numbers of TRACP^+^ multinucleated osteoclasts under control conditions was 29.8 ± 19.4/well in panel *D* and 36.0 ± 10.2/well in panel *E*. All values are the mean ± SD of at least three independent experiments. Original magnification ×10.

IL-6 is known to be a negative regulator of osteoblasts(23,30); therefore, we hypothesized that osteoblast differentiation and activity could be decreased by DMD sera. To address this hypothesis, we incubated human primary osteoblasts from healthy donors with 10% of DMD or healthy sera before assaying matrix mineralization and testing differentiation markers. Mineralized nodule formation was decreased in osteoblast cultures containing sera from DMD patients (Fig. 3*A*), whereas the histochemical and biochemical evaluation of ALP showed no modulation (Fig. 3*B*). In addition, the mRNA levels of the osteoblast transcription factor *osterix* and of the matrix protein *osteocalcin* evaluated by real-time RT-PCR were significantly decreased, whereas *Runx2*, which is known to be crucial for early osteoblast differentiation, was not affected (Supplemental Fig. S1). To assess whether these transcriptional effects were IL-6-dependent, we incubated the cells in the presence of an IL-6-blocking antibody and observed that this treatment prevented the decrease of *osterix* and *ostecalcin* mRNA induced by the DMD sera (Fig. 3*C*).

To better analyze how osteoblast differentiation and activity were affected by imbalanced circulating cytokines, we performed real-time RT-PCR arrays to evaluate the transcription levels of a panel of osteoblast differentiation markers. A decrease in many osteogenic factors in osteoblasts cultured with DMD sera versus control sera, in particular of various bone morphogenetic proteins (BMPs), was in agreement with the hypothesized impairment of osteoblast function (Supplemental Table S2). Taken together, these results suggest that osteoblasts are disturbed by DMD serum cytokine imbalance, especially at the late stage of differentiation, and that increased IL-6 may represent one of the mediators of the observed modulations.

### Effect of DMD sera on osteoclastogenesis

Cytokine imbalance in sera suggested that osteoclast formation also could be changed if peripheral blood precursors were exposed to DMD sera. To analyze this aspect, peripheral blood mononuclear cells from healthy donors were cultured in medium supplemented with 10% sera from DMD patients or normal subjects, 25 ng/mL of M-CSF, and a suboptimal sRANKL (0.5 ng/mL) for 14 days.(29) Interestingly, significant enhancement of osteoclastogenesis was noted with patients' sera compared with healthy sera (2.3-fold), as evidenced by quantification of TRACP^+^ multinucleated cells (Fig. 3*D*). Moreover, circulating mononuclear precursors from DMD patients showed a parallel higher osteoclast-forming ability than those from matched normal donors (1.4-fold; Fig. 3*E*), suggesting that imbalanced cytokines especially could affect the number of osteoclast precursors in DMD patients.

To explain how osteoclast formation could be enhanced notwithstanding the low circulating sRANKL/OPG ratio previously described, we hypothesized alterations of some other factors normally involved in the osteoblast-osteoclast cross-talk. To this end, we performed real-time RT-PCR arrays on human primary osteoblasts from healthy donors incubated with 10% of DMD or healthy sera for 48 hours to evaluate the transcription level of a cytokine panel. Among the few upregulated mRNAs (Table 5), *IL11* was the most increased (>4-fold), followed by *inhibin-βA*, *IL6*, and *TGFβ2*. However, IL-11 was not detectable in both DMD and healthy sera, whereas measurements of inhibin-βA and TGF-β2 revealed no difference between DMD and healthy sera (inhibin-βA: control 256.0 ± 61.0 pg/mL; DMD 237.0 ± 74.0 pg/mL; TGF-β2: control 123.0 ± 91.0 pg/mL; DMD 117.0 ± 54.0 pg/mL; differences not significant), thus pointing out that their effect could be exerted only locally in the bone microenvironment in response to systemic factors. On the contrary, the *IL6* mRNA increase in osteoblasts cultured with DMD sera was similar to the increase of its circulating level in patients (>2-fold).

**Table 5:** Cytokines Upregulated in Osteoblasts Treated With DMD Sera

Gene name	Gene symbol	Fold change	*p* Value
Interleukin 11	*IL11*	4.1 ± 0.13	.00003
Inhibin-βA	*INHBA*	3.1 ± 0.15	.03
Interleukin 6	*IL6*	2.2 ± 0.26	.003
Transforming growth factor β2	*TGFB2*	2.0 ± 0.24	.002

*Note:* Fold change represents the gene expression ratio between osteoblasts incubated with patient sera and control sera, respectively, and is expressed as mean ± SD of three independent experiments. *p* Value indicates the significance level.

Among the many downregulated cytokine mRNAs (Supplemental Table S3), we found several members of the IL-1 superfamily: *IL2*, *IL3*, *IL4*, and *TNFα⋅* This latter was claimed to be implicated in the inflammation of DMD patients(12) but in our assays appeared undetectable in the circulation of DMD patients (Table 4) and strongly downregulated in osteoblasts treated with the DMD sera (86% less compared with healthy sera; Supplemental Table S3).

**Fig. 4:**
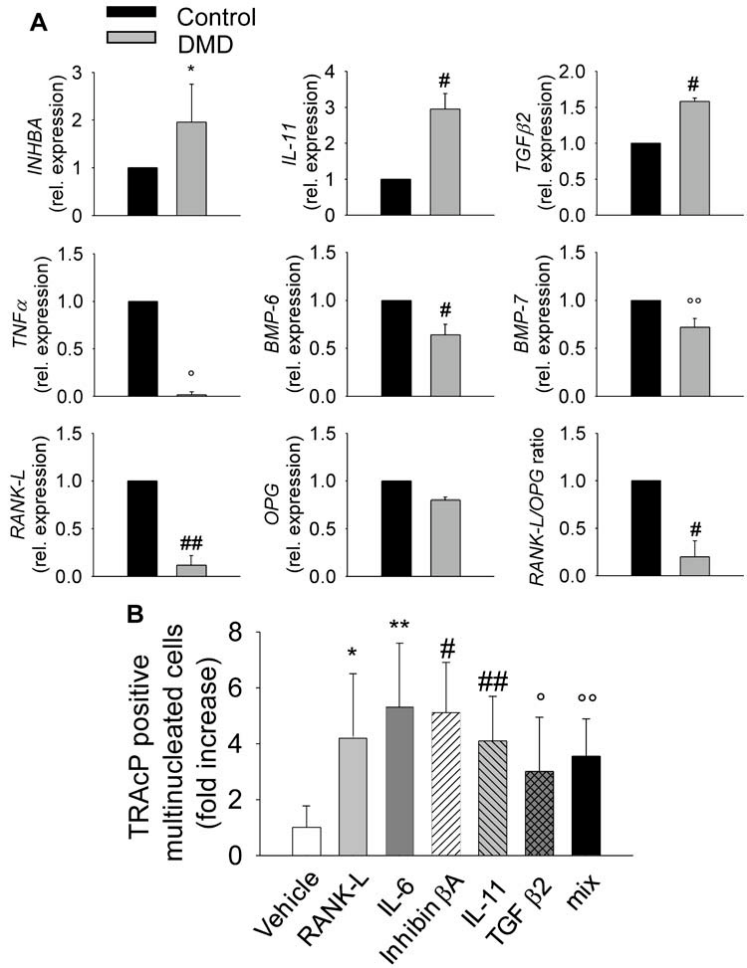
Cytokine expression and osteoclastogenesis assay. (*A*) Osteoblasts from healthy donors were incubated with sera from controls or DMD patients, as described in “Materials and Methods.” RNA was extracted and reverse transcribed, and cDNA was subjected to comparative real-time PCR using primer pairs and conditions specific for *inhibin-βA* (*INHBA*), *IL11*, *TGFβ2, TNFα, bone morphogenic protein 6* (*BMP6*)*, BMP7, RANKL*, and *OPG*. *RANKL/OPG* ratio is also shown. ^#^*p* = .02; ^°°^*p* = .03; *p = .04; ^°^*p* = .002; and ^##^*p* = .003 versus control. Values are normalized versus the housekeeping gene *GAPDH.* (*B*) Human peripheral blood mononuclear cells from healthy donors were cultured, as described in “Materials and Methods,” in the presence of M-CSF and 10 ng/mL of IL-6, IL-11, inhibin-βA, TGF-β2, or sRANKL as positive control. TRACP^+^ multinucleated cells were enumerated and expressed as fold increase of control. Average numbers of osteoclasts in control conditions (Vehicle) was 19.0 ± 6.0/well. **p = .02; *p = .04; ^#^*p* = .007; ^##^*p* = .003; and ^°^*p* = .0007 versus control. All values are the mean ± SD of three independent experiments.

Expression of selected genes from the RT-PCR array also was validated by standard real-time RT-PCR (Fig. 4*A*). In addition, by real-time RT-PCR we evaluated the transcriptional expression of RANKL and OPG, which were not included in the osteoblast cytokine array. We again observed downregulation of *RANKL* mRNA (88% less compared with healthy sera) and no significant change in *OPG* mRNA, with a subsequent decrease in the osteoblast RANKL/OPG ratio (80% less than in healthy sera; Fig. 4*A*), in agreement with the reduced protein ratio shown previously in *MDX* and DMD sera (Fig. 1 and Table 4).

To assess whether the earlier described most upregulated cytokines with osteoclastogenic potential were able to increase osteoclastogenesis in the absence of sRANKL, we performed healthy-donor peripheral blood mononuclear cell cultures incubated for 14 days in the presence of 25 ng/mL of M-CSF and 10 ng/mL of human recombinant IL-6, inhibin-βA, IL-11, or TGF-β2, alone or in combination, and compared the results with the same concentration of sRANKL. Induction of osteoclastogenesis was evident for all these cytokines relative to M-CSF alone (Fig. 4*B*). Interestingly, the effects of the four cytokines in combination did not appear to be additive, consistent with convergence on common intracellular pathways. Altogether, these results highlight that imbalanced osteoclastogenic cytokines could be responsible of enhanced osteoclast formation even in the event of reduced sRANKL/OPG levels.

### Treatment of bone organ cultures with IL-6-blocking antibody

**Fig. 5:**
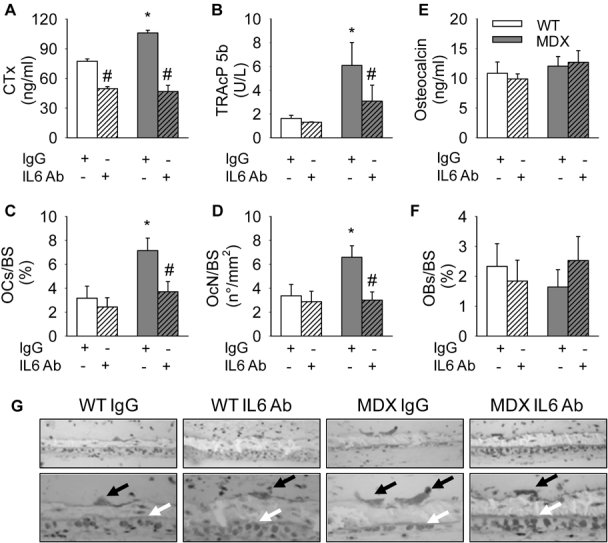
Effect of blocking IL-6 activity on murine calvarial bones in culture. Four-day-old CD1 mouse calvarial bones were cultured as described in “Materials and Methods” in the presence of a blocking antibody against IL-6 (IL-6 Ab) or an irrelevant IgG. ELISA assay for detection of (*A*) C-terminal telopeptide of type 1 collagen (CTX) and (*B*) TRACP-5b in conditioned medium. Histomorphometric evaluation of (*C*) osteoclast surface/bone surface (OcS/BS, %) and (*D*) osteoclast number/bone surface (OcN/BS, *n*/mm^2^). (*E*) ELISA assay for osteocalcin in conditioned medium. (*F*) Histomorphometric evaluation of osteoblast surface/bone surface (ObS/BS, %). (*G*) Coronal semithin sections of the calvarial bones stained with methylene blue/azure II. Original magnification ×20 (*upper panels*) and ×40 (*lower panels*). *Black arrows* = osteoclasts; *white arrows* = osteoblasts. Values are the mean ± SD of three calvarial bones per group.*p = .04 versus wild type with irrelevant IgG; ^#^*p* = .03 versus each group treated with irrelevant IgG.

To assess whether, among the imbalanced cytokines, circulating IL-6 played a dominant role in osteoclast formation and to further support a translational meaning of our observations, we cultured ex vivo calvarial bones excised from wild-type neonatal mice in the presence of 10% sera from wild-type or *MDX* mice with or without an IL-6-blocking antibody. We observed that the bone-resorption parameter CTX and the osteoclast parameter TRACP were increased significantly in the medium in the presence of *MDX* sera compared with wild-type sera, and in both conditions, treatment with the blocking antibody reduced these biomarkers (Fig. 5*A*, *B*). This was in agreement with the histomorphometric evaluation of the osteoclast surface and number (Fig. 5*C*, *D*, *G*). In this circumstance, as shown by osteocalcin detection in the medium (Fig. 5*E*) and histomorphometric evaluation in the sections (Fig. 5*F*, *G*), osteoblast parameters were not affected, possibly owing to the short-term treatment performed in our experiments. These results suggest an impact of systemic IL-6 on the osteoclast and bone-resorption parameters that could be blocked by anti-IL-6 treatment.

## Discussion

Altogether, our results suggest that the defective bone mass in DMD is secondary not only to the failure in muscular traction but also to the marked uncoupling of osteoclast and osteoblast activity potentially caused by imbalanced circulating and local cytokines, among which we point to IL-6 as a crucial systemic mediator of bone loss.(23) These results are in keeping with a recent report by Nakagaki and colleagues,(31) who demonstrated reduced bone mass and altered bone mechanical and biochemical properties in *MDX* mice at 21 days of life, when histologic sections and Evans blue staining showed no muscle fiber damage, but are in disagreement with Montgomery and colleagues,(32) who evidenced increased femoral BMD in 4-month-old *MDX* mice.

Inflammation and inflammatory cells present in DMD muscles play an important role in generating atrophy and in inhibiting regeneration, therefore promoting progression of muscle damage,(33) but the exact inflammatory mechanisms and the pivotal inflammatory molecules involved so far are not known. There is now evidence that the bone tissue is also significantly affected in DMD and that low bone mass and increased frequency of fractures may be complications even at an early stage of the disease.(18–22,34) Many cytokines and other local regulators are involved in the control of bone remodeling with a high degree of redundancy,(35,36) and many cell types take part in this process, including osteoblasts, osteoclasts, inflammatory cells, and cells of the immune system.(37,38)

Skeletal muscle has been identified recently as an endocrine organ producing a number of “myokines“ belonging to different cytokine families, and IL-6 was discovered as a myokine because it systemically increases during physical exercise.(39,40) Interestingly, IL-6 also has been observed to be increased in dystrophic muscles(41) and therefore may represent one of the mediators of the bone phenotype in DMD. IL-6 plays a crucial role in bone metabolism, especially in nonphysiologic conditions. In fact, it has been shown that chronically increased systemic levels of IL-6 induce significant bone loss in growing *IL-6* transgenic mice(23) and that chronically elevated IL-6 is associated with bone loss in postmenopausal and juvenile osteoporosis.(42,43) This cytokine also has been implicated in the pathogenesis of osteolysis associated with Paget disease,(44) multiple myeloma,(45,46) Gorham-Stout disease,(47) and juvenile idiopathic arthritis.(48)

Our work demonstrates that IL-6 is involved in the bone phenotype of both DMD patients and *MDX* mice. An extensive analysis of osteogenic markers in osteoblasts exposed to DMD sera showed an overall downregulation of many transcripts, consistently with the poor ability of these cells to mineralize the bone matrix. Specifically, bone morphogenetic protein 4 (BMP-4) and BMP-6, which showed about 60% and 80% decrease, respectively, are known to strongly stimulate osteoblastogenesis,(49,50) whereas *osterix* and *osteocalcin* are osteoblast genes implicated in osteoblast maturation and bone formation.(51) Among the other modulated transcripts, most are not yet clearly correlated with osteoblast differentiation. Nevertheless, amelogenin and enamelin, for example, which are known to be crucial in dental matrix maturation,(52,53) seem to correlate with the lower ability of osteoblasts to fully comply with their functions. Taken together, these data suggest an effect of DMD circulating factors on late stage of osteoblast differentiation and subsequent matrix defects. The blunting effect of IL-6-neutralizing antibody was observed in these cultures, supporting a role of this cytokine in the impaired activity of osteoblasts.

In addition, osteoclast precursors were affected by DMD and *MDX* sera. In both circumstances, we observed an increase of osteoclastogenesis. This result is again in agreement with the increase in circulating IL-6 because it was able alone to induce osteoclastogenesis in vitro in the absence of sRANKL. Moreover, an extensive analysis of many cytokines expressed by osteoblasts exposed to DMD sera revealed a notable upregulation of several local factors potentially able to stimulate osteoclastogenesis, which, however, were not observed to be changed systemically in the circulation. Among them, IL-11 is already known to promote osteoclast maturation and bone resorption in bone marrow cultures,(54,55) and inhibin-βA was associated recently with induction of osteoclastogenesis in the absence of RANKL.(56) Interestingly, among the downregulated cytokines, TNF-α, which could be involved in the inflammation state that characterizes DMD muscles,(12) was not detectable in DMD patient sera. Also, in *MDX* mice, the osteopenic/osteoporotic bone phenotype appeared to be caused by an imbalance between osteoblast and osteoclast activity. In fact, especially the osteoclast lineage also was affected in calvaria, in which mechanical loading is negligible, so confirming a potential involvement of circulating cytokines affecting these cells. Among these potential mediators, IL-6 appears to be a pivotal factor not only in humans but also in mice because an IL-6-blocking antibody prevented the increase in osteoclast formation induced by *MDX* sera in calvarial organ cultures.

The role of IL-6 in the DMD bone disease may open new therapeutic perspectives based on anti-IL-6-receptor therapy. An IL-6 receptor inhibitor (humanized monoclonal antibody to the IL-6 receptor, tocilizumab) is already in clinical trials for childhood inflammatory diseases.(57) Unfortunately, to the best of our knowledge, the only commercially available anti-murine IL-6 receptor–neutralizing antibody is from rat and can be used solely in mice in short-term experiments because it stimulates an immune response and is rapidly inactivated. This prevented us from performing long-term treatments necessary to unveil a positive effect on bone in *MDX* mice. However, and consistent with our hypothesis, treatment with commercially available IL-6-blocking antibodies prevented the effects of dystrophic sera both on mouse and human bone. Therefore, we deem that this observation could have a translational impact and could open a new avenue for the treatment of bone disease in DMD patients with anti-IL-6 therapy.

In conclusion, we believe that our work provides an understanding of the molecular and cellular aspects of bone loss in DMD and a rationale for new therapies to maintain the bone structure and improve bone strength, which may, in turn, contribute to a better quality of life in DMD patients.
