# Supplementary material for: Mechanisms Inducing Low Bone Density in Duchenne Muscular Dystrophy in Mice and Humans
Source: J Bone Miner Res. 2011 Apr 20;26(8):1891–903. doi: 10.1002/jbmr.410 (PMC3150693; doi:10.1002/jbmr.410)
Supplement: Supplementary file 1 [file jbmr0026-1891-SD1.doc]

**SUPPLEMENTAL MATERIAL**

**Supplemental Figure 1.** Osteoblasts were grown in the presence of 10% sera pooled from healthy controls or DMD patients. After 48 hours RNA was extracted and reverse transcribed, and cDNA was subjected to comparative real time PCR using primer pairs and conditions specific for *Osterix (OSX)*, *Osteocalcin (OCN)* and *Runt-related transcription factor 2* *(RUNX-2).* #p=0.004 and *p=0.0005 vs.

| **Supplemental Table 1. Primer pairs employed for real time RT-PCR** | | |
| --- | --- | --- |
| Cytokines | Forward | Reverse |
| *GAPDH*  *IL-6*  *TNF-α*  Inhibin βA  *RANK-L*  *OPG*  *IL-11*  *TGFβ2*  *RUNX-2*  *OSTEOCALCIN*  *OSTERIX*  *BMP-6*  *BMP-7* | 5’- AGGTCCACCACTGACACGTT-3’  5’- CTCAGGGCTGAGATGCCG-3’  5’-CCTGTGAGGAGGACGAACAT-3’  5’-TATAGTGTTAACCAAGATTC-3’  5’-AGAGCGCAGATGGATCCTAA-3’  5’-GTGTCTTGGTCGCCATTTTT-3’  5’-CAGGGTGACTTGTGGACCT-3’  5’-TGCTTTGGCTTTCTGGTTCT-3’  5’-TGGCTGCATTGAAAAGACTG-3’  5’-AGCAAAGGTGCAGCCTTTGT-3’  5’-CGTAGTGCGAACCGAGGTCA-3’  5’-CTCGGGGTTCATAAGGTGAA-3’  5’-TGTGATCCCTCTTGGTGTGA-3’ | 5’- CTGCACCACCAACTGCTTAG-3’  5’- TCTCCACAAGCGCCTTGC-3’  5’-AGGCCCCAGTTTGAATTCTT-3’  5’-GCTAGTGGCTACCAAGGTTA-3’  5’-TTCCTTTTGCACAGCTCCTT-3’  5’-GGCAACACAGCTCACAAGAA-3’  5’-GCAATCTGAGGTTCACTGCAA-3’  5’-TTTGTTTGTGGTGCAGTGGT-3’  5’-TTTGCACTGGGTCATGTGTT-3’  5’-GCGCCTGGGTCTCTTCACT-3’  5’-GGTAGTGCGAACCAAGGCCA-3’  5’-CTTACGACAAGCAGCCCTTC-3’  5’-CAGGTTGAAGGAAAGCAAGC-3’ |

PCR conditions were 40 cycles: 94° C for 45 s, 60° C for 45 s and 72° C for 45 s.

**Supplemental Table 2. Osteogenic genes regulated in osteoblasts treated with DMD sera**

| *Gene name* | *Gene symbol* | *Fold change* | *P value* |
| --- | --- | --- | --- |
| Fibroblast growth factor 2 | *FGF2* | 1.35 ± 0,02 | 0.00001 |
| Alkaline phosphatase, liver/bone/kidney | *ALPL* | 0.81 ± 0,09 | 0.03 |
| Ameloblastin (enamel matrix protein) | *AMBN* | 0.27 ± 0,37 | 0.03 |
| Amelogenin, Y-linked | *AMELY* | 0.10 ± 0,10 | 0.0001 |
| Bone gamma-carboxyglutamate (gla) protein (osteocalcin) | *BGLAP* | 0.52 ± 0,26 | 0.03 |
| Bone morphogenetic protein 3 | *BMP3* | 0.16 ± 0,14 | 0.0006 |
| Bone morphogenetic protein 4 | *BMP4* | 0.42 ± 0,34 | 0.04 |
| Bone morphogenetic protein 5 | *BMP5* | 0.15 ± 0.12 | 0.0002 |
| Bone morphogenetic protein 6 | *BMP6* | 0.23 ± 0,10 | 0.0002 |
| Bone morphogenetic protein 7 | *BMP7* | 0.11 ± 0.16 | 0.0007 |
| Bone morphogenetic protein 8B | *BMP8B* | 0.19 ± 0.08 | 0.0005 |
| Calcitonin receptor | *CALCR* | 0.37 ± 0,32 | 0.03 |
| Collagen, type IV, α 3 | *COLL4A3* | 0.16 ± 0,04 | 0.000003 |
| Colony stimulating factor 2 (granulocyte-macrophage) | *CSF2* | 0.08 ± 0,13 | 0.0002 |
| Dentin matrix acidic phosphoprotein | *DMP1* | 0.30 ± 0,43 | 0.05 |
| Enamelin | *ENAM* | 0.10 ± 0,09 | 0.0007 |
| Fibroblast growth factor 3 | *FGF3* | 0.05 ± 0,07 | 0.00002 |
| Fms-related tyrosine kinase 1 | *FLT1* | 0.20 ± 0,16 | 0.0009 |
| Growth differentiation factor 10 | *GDF10* | 0.22 ± 0,33 | 0.02 |
| Intercellular adhesion molecule 1 (CD54) | *ICAM1* | 0.44 ± 0,30 | 0.03 |
| Insulin-like growth factor 2 (somatomedin A) | *IGF2* | 0.44 ± 0,29 | 0.03 |
| Matrix metallopeptidase 8 | *MMP8* | 0.06 ± 0,03 | 4E-07 |
| Matrix metallopeptidase 9 | *MMP9* | 0.36 ± 0,34 | 0.03 |
| Msh homeobox homolog 1 (Drosophila) | *MSX1* | 0.50 ± 0,30 | 0.05 |
| Scavenger receptor class B, member 1 | *SCARB1* | 0.62 ± 0,12 | 0.006 |
| SMAD, mothers against DPP homolog 1 | *SMAD1* | 0.72 ± 0,10 | 0.01 |
| Statherin | *STATH* | 0.13 ± 0,12 | 0.0002 |

*Fold change* represents the gene expression ratio between osteoblasts incubated with patients’ sera and controls’ sera, respectively, and is expressed as mean ± SD of 3 independent experiments. *P value* indicates the significance level.

| **Supplemental Table 3. Down-regulated cytokine genes in osteoblasts treated with DMD sera** | | | |
| --- | --- | --- | --- |
| *Gene name* | *Gene symbol* | *Fold change* | *P value* |
| Family with sequence similarity 3, member B | *FAM3B* | 0.04 ± 0.06 | 0.0001 |
| Fas ligand (TNF superfamily, member 6) | *FASLG* | 0.13 ± 0.19 | 0.001 |
| c-fos induced growth factor | *FIGF* | 0.30 ± 0.43 | 0.05 |
| Growth differentiation factor 10 | *GDF10* | 0.05 ± 0.01 | 0.00000001 |
| Growth differentiation factor 2 | *GDF2* | 0.02 ± 0.02 | 0.00000007 |
| Growth differentiation factor 3 | *GDF3* | 0.04 ± 0.06 | 0.000007 |
| Growth differentiation factor 5 | *GDF5* | 0.41 ± 0.10 | 0.0005 |
| Myostatin | *MSTN* | 0.25 ± 0.27 | 0.008 |
| Growth differentiation factor 9 | *GDF9* | 0.28 ± 0.22 | 0.005 |
| Interferon α 2 | *IFNA2* | 0.14 ± 0.22 | 0.002 |
| Interferon α 4 | *IFNA4* | 0.12 ± 0.15 | 0.0005 |
| Interferon α 5 | *IFNA5* | 0.19 ± 0.30 | 0.01 |
| Interferon α 8 | *IFNA8* | 0.08 ± 0.10 | 0.00008 |
| Interferon β 1 | *IFNB1* | 0.18 ± 0.25 | 0.005 |
| Interferon γ | *IFNG* | 0.10 ± 0.15 | 0.0005 |
| Interferon κ | *IFNK* | 0.08 ± 0.10 | 0.00008 |
| Interleukin 10 | *IL10* | 0.04 ± 0.04 | 0.000001 |
| Interleukin 12B | *IL12B* | 0.09 ± 0.08 | 0.00004 |
| Interleukin 13 | *IL13* | 0.14 ± 0.04 | 0.000004 |
| Taxilin alfa | *TXLNA* | 0.33 ± 0.41 | 0.05 |
| Interleukin 17° | *IL17A* | 0.09 ± 0.09 | 0.00007 |
| Interleukin 17C | *IL17C* | 0.05 ± 0.02 | 0.0000002 |
| Interleukin 25 | *IL25* | 0.03 ± 0.02 | 0.00000004 |
| Interleukin 18 | *IL18* | 0.53 ± 0.10 | 0.001 |
| Interleukin 19 | *IL19* | 0.04 ± 0.06 | 0.000009 |
| Interleukin 1 α | *IL1A* | 0.11 ± 0.07 | 0.00002 |
| Interleukin 1 β | *IL1B* | 0.21 ± 0.08 | 0.0005 |
| Interleukin 1 family, member 10 | *IL1F10* | 0.08 ± 0.06 | 0.00001 |
| Interleukin 1 family, member 5 | *IL1F5* | 0.06 ± 0.06 | 0.00001 |
| Interleukin 1 family, member 6 | *IL1F6* | 0.12 ± 0.19 | 0.001 |
| Interleukin 1 family, member 7 | *IL1F7* | 0.09 ± 0.10 | 0.0001 |
| Interleukin 1 family, member 8 | *IL1F8* | 0.05 ± 0.06 | 0.00001 |
| Interleukin 1 family, member 9 | *IL1F9* | 0.05 ± 0.07 | 0.00002 |
| Interleukin 2 | *IL2* | 0.05 ± 0.07 | 0.00002 |
| Interleukin 20 | *IL20* | 0.08 ± 0.09 | 0.00005 |
| Interleukin 21 | *IL21* | 0.15 ± 0.20 | 0.002 |
| Interleukin 22 | *IL22* | 0.12 ± 0.08 | 0.00005 |
| Interleukin 24 | *IL24* | 0.12 ± 0.06 | 0.00001 |
| Interleukin 3 | *IL3* | 0.03 ± 0.03 | 0.0000007 |
| Interleukin 4 | *IL4* | 0.04 ± 0.05 | 0.00004 |
| Interleukin 5 | *IL5* | 0.27 ± 0.21 | 0.004 |
| Interleukin 7 | *IL7* | 0.37 ± 0.30 | 0.02 |
| Interleukin 9 | *IL9* | 0.15 ± 0.12 | 0.0002 |
| Inhibin, α | *INHA* | 0.21 ± 0.14 | 0.0006 |
| Left-right determination factor 2 | *LEFTY2* | 0.06 ± 0.07 | 0.00003 |
| Lymphotoxin α | *LTA* | 0.04 ± 0.04 | 0.000002 |
| Lymphotoxin β | *LTB* | 0.03 ± 0.03 | 0.0000006 |
| Nodal homolog (mouse) | *NODAL* | 0.17 ± 0.05 | 0.000006 |
| Transforming grow factor α | *TGFA* | 0.35 ± 0.36 | 0.03 |
| Tumor necrosis factor α | *TNF* | 0.14 ± 0.17 | 0.001 |
| Tumor necrosis factor (ligand) superfamily, member 11 | *TNFSF11* | 0.35 ± 0.22 | 0.0006 |
| Tumor necrosis factor (ligand) superfamily, member 12 | *TNFSF12* | 0.63 ± 0.11 | 0.005 |
| Tumor necrosis factor (ligand) superfamily, member 13B | *TNFSF13B* | 0.49 ± 0.07 | 0.0002 |
| Tumor necrosis factor (ligand) superfamily, member 14 | *TNFSF14* | 0.07 ± 0.06 | 0.00001 |

*Fold change* represents the gene expression ratio between osteoblasts incubated with patients’ sera and controls’ sera, respectively, and is expressed as mean ± SD of 3 independent experiments. *P value* indicates the significance level.
